# T Wave Inversion: A Screening Tool for Rapidly Differentiating Acute Coronary Syndrome and Pulmonary Embolism

**DOI:** 10.7759/cureus.66950

**Published:** 2024-08-15

**Authors:** Saeed Namjoo, Morteza Azari, Farnaz Kamali, Mahsa Moosavi, Mahdi Rahmanian, Hamed Bazrafshan Drissi

**Affiliations:** 1 Department of Internal Medicine, Shiraz University of Medical Sciences, Shiraz, IRN; 2 Cardiovascular Research Center, Shiraz University of Medical Sciences, Shiraz, IRN; 3 Department of Endocrinology, Shiraz University of Medical Sciences, Shiraz, IRN; 4 Department of Mathematics and Statistics, University of Regina, Saskatchewan, Regina, CAN; 5 Department of Cardiology, Shiraz University of Medical Sciences, Shiraz, IRN

**Keywords:** pulmonary embolism, acute coronary syndrome, electrocardiography, t wave characteristics, morbidity, mortality

## Abstract

Introduction

Acute coronary syndrome (ACS) and acute pulmonary embolism (PE) are life-threatening conditions with similar clinical presentations. As current diagnostic tools, such as computed tomography pulmonary angiography, for distinguishing between these two conditions are time-consuming and may not be available in all settings, we tried in this study to devise a diagnostic tool based on electrocardiography to distinguish between ACS and acute PE based on T wave features.

Methods

Medical records of patients with diagnoses of ACS and acute PE, who were referred to three hospitals affiliated with Shiraz University of Medical Sciences, Shiraz, Iran, from March 2019 to March 2021, were evaluated. One expert cardiologist read patients’ electrocardiograms (ECGs). All ECGs were recorded at the standard 25 mm/s and 10 mm/mV. The sum of T wave inversion or TWI (mV) in consecutive leads, including anterior leads (V1, V2, V3, and V4), inferior leads (II, III, aVF), and lateral leads (I, aVL, V5, and V6) were calculated to estimate the cut-off points used to differentiate ACS versus acute PE. The receiver operating characteristic (ROC) curve was used to estimate the diagnostic accuracy of T wave changes. The Youden index was used to calculate the optimum cut-offs for sensitivity and specificity.

Results

Of 151 patients with a mean age of 55.44±12.88 years, 74 were in the acute PE and 77 were in the ACS groups. The results showed that the TWI sum in anterior leads >1.2 mV (P<0.001), in lateral leads >0.9 mV (P<0.001), in anterior-to-inferior leads ratio >12 (P<0.001), and V_4_/V_1_ leads ratio >4 (P<0.001) rules out acute PE. Anterior-to-lateral TWI ratio (AUC=0.807, sensitivity=70.3%, specificity=10%) was significantly distinctive among ACS and acute PE patients.

Conclusion

TWI sum in anterior leads >1.2 mV, in lateral leads >0.9 mV, in anterior-to-inferior leads ratio >12, and in V_4_/V_1_ leads ratio >4 rules out acute PE. The anterior-to-lateral TWI ratio obtained from patients’ ECG was significantly distinctive among the patients and can be used as a screening tool.

## Introduction

Acute pulmonary embolism (PE) is a life-threatening medical emergency that occurs because of the emboli, resulting in obstruction of the pulmonary artery or its branches. It is the third most common cardiovascular disease following myocardial infarction and stroke, which leads to remarkable mortality and morbidity [1]. Improvements in the availability and accuracy of diagnostic tests in the last 20 years and the introduction of anticoagulants have resulted in a tremendous decrease in the mortality rate of acute PE while its incidence rate has doubled, owing to the increased diagnostic accuracy [2].

The most prevalent symptoms in patients with acute PE are non-specific and similar to other cardiac conditions and include dyspnea, chest pain, and cough. Therefore, clinical manifestations are not sufficient for definite diagnosis and differentiation from acute coronary syndrome (ACS), the most common cardiac condition with similar clinical symptoms. Diagnosis is even more challenging in patients with stable hemodynamics. Probability assessment tests, such as D-dimer testing and definitive diagnostic imaging like computed tomography (CT), are suggested to diagnose stable patients suspected of acute PE and echocardiography or venous compression ultrasound for unstable patients [3]. These assessments are time-consuming and require multidisciplinary teams for accurate diagnosis and appropriate treatment [4]. Therefore, identifying a more straightforward and quicker diagnostic tool can help with early diagnosis and treatment and thus improve patients' outcomes. Electrocardiography (ECG) is a simple tool generally performed as a routine examination for all patients with cardiac or respiratory symptoms in the emergency department (ED). Despite the low diagnostic accuracy of ECG for acute PE, a set of abnormal ECG findings in ECG have been related to the associated complications of acute PE such as hemodynamic decompensation, right ventricle (RV) dysfunction, increased mean pulmonary artery pressure, in-hospital complications, cardiogenic shock, and death [5]. Prognostic value has been reported for abnormal ECG findings, including S1Q3T3, low QRS voltage, changes in ST segment, right bundle branch block [6,7], and T wave inversion (TWI) in different leads [8,9]. Some studies have suggested that different distributions of TWI in leads III and V1 can differentiate acute PE from ACS [10,11].

Based on a previous study on the comparison of TWI in ACS and acute PE [12] and the fact that T inversion is a common finding among PE and ACS, we aimed to try developing a diagnostic tool based on ECG to distinguish these two clinical conditions. Also, considering the clinical value of differentiating acute PE and ACS in the emergency department (ED), the ease and accessibility of this test, and the previous evidence on this issue, in this study, we aimed to distinguish between ACS and acute PE based on T wave features that can aid in rapid and accurate differentiation of these two conditions and enhance patient outcomes.

## Materials and methods

Study design

In this cross-sectional study, medical records of patients who were referred to Shahid Faghihi, Nemazee, and Al-Zahra Hospitals affiliated with Shiraz University of Medical Sciences, Shiraz, Iran, from March 2019 to March 2021, were evaluated, and those diagnosed with non-STEMI ACS or acute PE were enrolled in the study. A definite diagnosis of acute PE was made by CT angiography of the pulmonary artery, which an expert radiologist reported. If a central filling defect within a pulmonary artery surrounded by contrast material or an eccentric or mural filling defect was observed, the diagnosis of acute PE was made [13]. Based on the American Heart Association and the European Society of Cardiology, acute PE was classified as high risk (presence of hypotension, systolic BP < 90 mmHg, or drop of ≥ 40 mmHg for at least 15 minutes), intermediate risk (presence of right ventricular strain, dilation, or dysfunction), and low risk (do not meet criteria for intermediate risk).

Patients who were referred with new-onset symptoms at rest or recently worsened symptoms who had a high HEART (history, electrocardiogram, age, risk factors, and troponin) score (7 to 10 points) or medium HEART score (4 to 6 points) accompanied by a positive coronary CT angiography were considered ACS. Also, those who had ≥70% main coronary involvement in angiography were included in this study. Patients with Q-wave myocardial infarction (MI), decreased left ventricle ejection fraction on echocardiography, and ST-segment elevation MI (STEMI) were excluded.

G* power software (https://www.psychologie.hhu.de/arbeitsgruppen/allgemeine-psychologie-und-arbeitspsychologie/gpower) was used to calculate the minimum sample size. Based on a previous similar study, the mean magnitude of negative T was equal to 4.6 mm for ACS and 2.7 mm for precordial leads, so the effect size was calculated at 0.92. Considering α = 0.05 and study power = 0.9, and using the equation of comparing two means in two independent groups, the minimum sample size was calculated at 42 [12].

Demographic and clinical characteristics of the patients, including smoking, history of hypertension (HTN), and hyperlipidemia, were recorded from their medical records. Blood pressure measured at >140/90 mmHg or using anti-hypertensive medications was considered HTN. Serum level of cardiac troponin T I (cTnI), measured until 1 hour after admission to ED, was considered, and values ≥100 were considered positive. The ECG, taken in the ED, was evaluated and read by one expert cardiologist, and the presence of TWI >1 mm in at least two consecutive leads was recorded. All ECGs were recorded at the standard 25 mm/s and 10 mm/mV. In addition to the distribution and magnitude of TWI in limb and precordial leads, the peak of the negative T wave was also measured. The sum of T wave inversion (mV) in consecutive leads, including anterior leads (V1, V2, V3, and V4), inferior leads (II, III, aVF), and lateral leads (I, aVL, V5, and V6) were calculated to estimate the cut-off points used to differentiate ACS versus acute PE. ECG findings related to acute PE were also recorded, including P pulmonale, S1S2S3 pattern, S1Q3T3 pattern, and right axis deviation.

Patients with any condition that hindered accurate evaluation of ST-T change on ECG, such as complete left bundle branch block, hypertrophic cardiomyopathy, left ventricular hypertrophy, ventricular pacing, electrolyte abnormality, and metabolic diseases like severe anemia, were excluded. Also, a history of cardiac diseases that would influence the ECG was excluded, such as a history of revascularization, left ventricular hypertrophy, heart failure, and using digoxin. These data were obtained from patients' medical records.

The study protocol was approved by the Ethics Committee of Shiraz University of Medical Sciences (IR.SUMS.MED.REC.1399.320).

Statistical analysis

The collected data were input into the statistical software IBM SPSS Statistics for Windows version 21.0 (IBM Corp. 2012. Armonk, NY: IBM Corp.). Mean±standard deviation (SD) and frequency (percentage) were used to describe the data. Since we had a large sample size (>50), the normal distribution of variables was tested using the Kolmogorov-Smirnov test. TWI measures did not follow a normal distribution pattern. In order to compare T wave characteristics between the two groups, the Mann-Whitney U test was used; also, for comparing the values among three subgroups of acute PE type, the Kruskal Wallis test was used. For categorical variables, the chi-square test was used. To compare patients' age, blood pressure, and heart rate among the two groups, an independent sample T-test was used. The receiver operating characteristic (ROC) curve was also drawn. The area under the curve (AUC) with its sensitivity and specificity using the Youden index was calculated to estimate the diagnostic accuracy of T wave features in different ECG leads. P values (P) less than 0.05 were considered statistically significant in all tests. AUC greater than 0.6 was considered discriminative between ACS and acute PE; however, TWI measures with AUC greater than 0.8 were considered suitable for use as clinical tools.

## Results

Of 151 participants with a mean age of 55.44±12.88 years, 74 patients had acute PE, and 77 had ACS. The baseline characteristics of the groups are shown in Table 1. The mean systolic blood pressure of patients with ACS was significantly higher (P<0.001) while the mean pulse rate of patients with acute PE was higher (P<0.001) (Table 1). The ACS group had a higher frequency of smokers (57.1% vs. 37.8%; P=0.018) and positive cTnI (90.9% vs. 32.4%; P<0.001). Among patients with acute PE, 89.2% presented with dyspnea while 54.5% of patients with ACS presented with chest pain. Only 1.4% of acute PE patients had chest pain.

**Table 1:** Comparison of demographic and clinical characteristics between the two study groups *The results of the chi-square test; **The results of the independent sample T-test; DM: diabetes mellitus; HTN: hypertension; SD: standard deviation; N: number

Variable	Categories	Total	Acute pulmonary embolism	Acute coronary syndrome	P value^*^
Sex, N (%)	Male	96 (63.6)	36 (48.6)	60 (77.9)	0.001
	Female	55 (36.4)	38 (51.6)	17 (22.1)	
Age (years), mean±SD		55.44±12.88	55.67±18.30	59.54±11.52	0.12^**^
Systolic blood pressure, mean±SD		119.78±18.16	113.89±16.25	125.45±18.19	<0.001^**^
Pulse rate, mean±SD		90.39±14.11	97.11±14.50	83.93±10.24	<0.001^**^
Underlying diseases, N (%)	DM	43 (28.5)	16 (21.6)	27 (35.1)	0.048
	HTN	49 (32.5)	27 (36.5)	22 (28.6)	
	Hyperlipidemia	3 (2)	3 (4.1)	0	
	DM & HTN	23 (15.2)	9 (12.2)	14 (18.2)	
	None	33 (21.9)	19 (25.7)	14 (18.2)	
Smoker, N (%)	Yes	72 (47.7)	28 (37.8)	44 (57.1)	0.018
	No	97 (52.3)	46 (62.2)	33 (42.9)	
Presenting symptoms, N (%)	Dyspnea alone	66 (43.7)	66 (89.2)	0	<0.001
	Chest discomfort alone	43 (28.5)	1 (1.4)	42 (54.5)	
	Both	42 (27.8)	7 (9.5)	35 (45.5)	
Troponin-I, N (%)	Positive	94 (62.3)	24 (32.4)	70 (90.9)	<0.001
	Negative	57 (37.7)	50 (67.6)	7 (9.1)	

**Figure 1:**
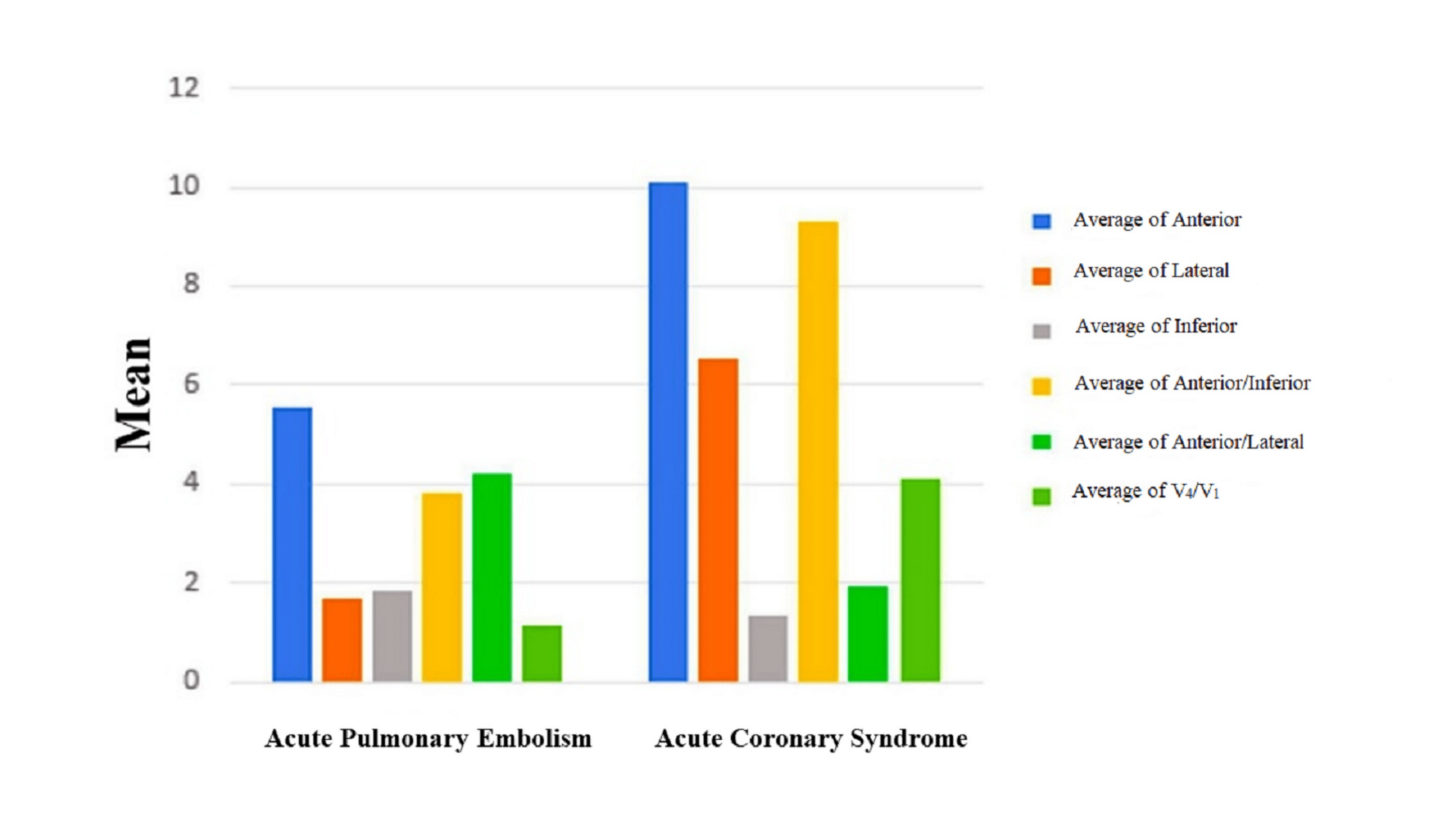
The mean depth of the T wave in precordial leads in patients with acute pulmonary embolism and acute coronary syndrome

The ACS group had a more significant TWI sum in anterior leads (P<0.001), lateral leads (P<0.001), anterior-to-inferior ratio (P<0.001), and V4/V1 ratio (P<0.001), and a lower sum in inferior leads (P<0.001) and anterior-to-lateral ratio (P<0.001) (Table 2). The results showed that the TWI sum in anterior leads >1.2 mV (P<0.001), in lateral leads >0.9 mV (P<0.001), in anterior-to-inferior leads ratio >12 (P<0.001), and V4/V1 leads ratio >4 (P<0.001) rules out acute PE. Figure 1 depicts these differences.

**Table 2:** Comparing the mean depth of the T wave in precordial leads between the two study groups *The results of the Mann Whitney U test; all values are reported as mean±SD

Variable	Acute pulmonary embolism	Acute coronary syndrome	P value^*^
Anterior lead	5.55±2.79	10.08±5.64	<0.001
Lateral lead	1.70±1.44	6.54±4.55	<0.001
Inferior lead	1.84±1.20	1.35±1.17	<0.001
Anterior/inferior	3.83±2.45	9.30±5.93	<0.001
Anterior/lateral	4.19±2.37	1.91±1.41	<0.001
V_4_/V_1_	1.12±0.89	4.09±2.44	<0.001

Among the patients with acute PE, 13 (17.6%) had the high-risk form of the disease, 41 (55.4%) had the intermediate-risk form, and 20 (27%) had the low-risk form. T wave features were not distinctive among acute PE subgroups (Table 3).

**Table 3:** The effect of disease type on the mean values of the T wave *The results of the Kruskal Wallis test; all values are reported as mean±SD; N: Number

Variable	Total	High-risk disease (N=13)	Intermediate-risk disease (N=41)	Low-risk disease (N=20)	P value^*^
Anterior/inferior	3.83±2.45	3.73±2.27	3.47±2.47	4.64±2.44	0.128
Anterior/lateral	4.19±2.37	4.43±2.37	3.99±2.55	4.45±2.04	0.364
V_4_/V_1_	1.12±0.89	1.15±0.87	0.92±0.74	1.49±1.10	0.131

**Figure 2:**
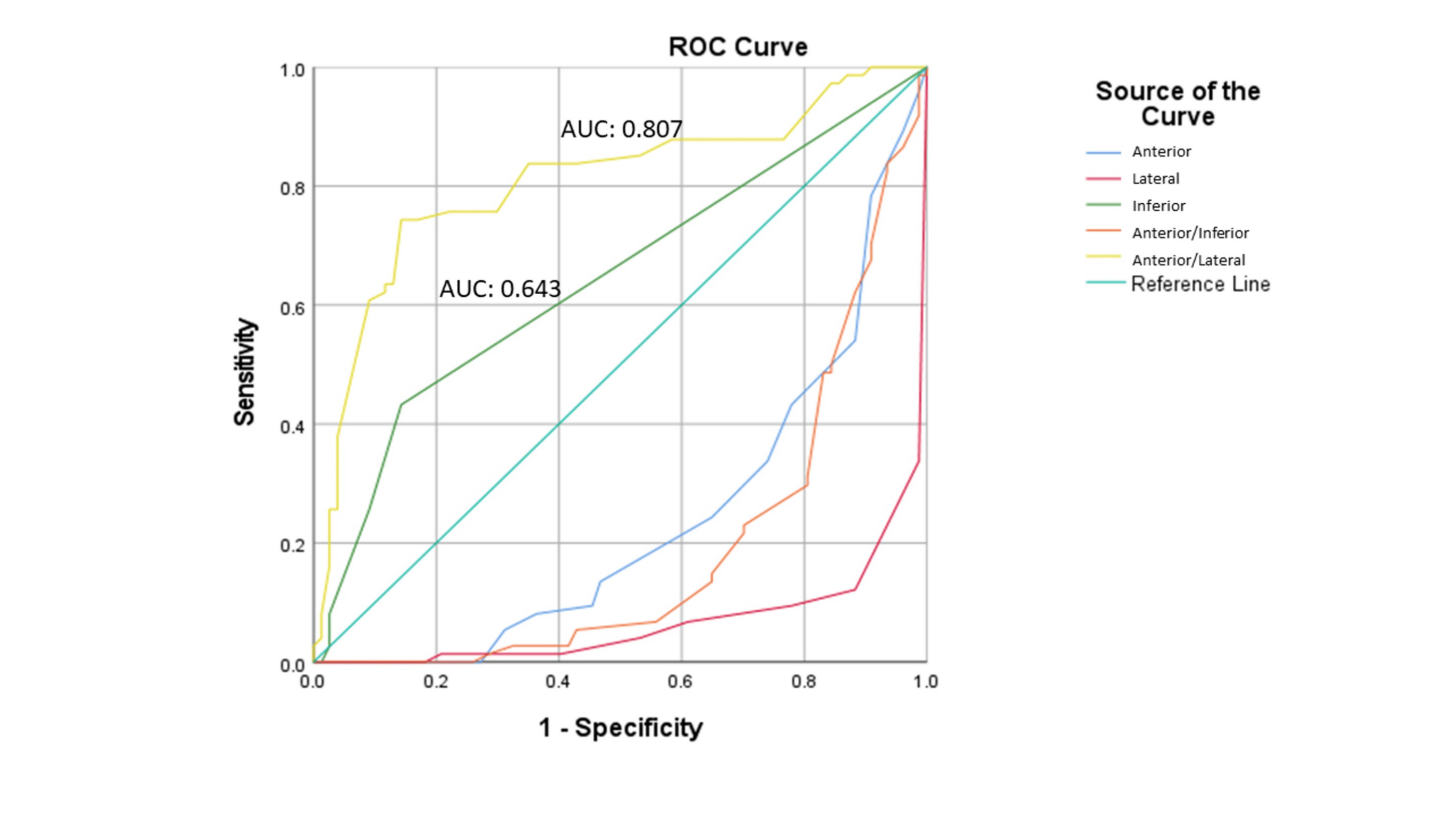
The ROC curve analysis for estimating the diagnostic accuracy of different electrocardiogram leads ROC curve: receiver operating characteristic curve; AUC: area under the curve

The ratio of the anterior-to-lateral TWI sum (AUC=0.807) and the TWI sum in inferior leads (AUC=0.643) had acceptable diagnostic accuracy for differentiation of acute PE and ACS while the other leads were below the reference line (Figure 2). However, the AUC of the anterior leads was 0.238, the anterior-to-inferior TWI ratio was 0.191, and the lateral lead was 0.068. Only the ratio of anterior-to-lateral TWI sum (AUC=0.807, sensitivity=70.3%, specificity=10%) was sensitive enough to be used as a screening tool to differentiate acute PE and ACS.

## Discussion

The present study compared the demographic and clinical characteristics and ECG findings between the patients with ACS and acute PE, and the results showed significant differences in the TWI sum in all precordial leads. Based on previous research, TWI in patients with acute PE can be predictive, especially when occurring in >5 leads [8]. The mechanism of TWI in patients with acute PE has been attributed to acute cor pulmonale with rapid overload and enlargement of RV. RV dysfunction and ischemia may impair the overall perfusion of cardiac myocardium [8,14]. TWI in patients with ACS, especially deeply inverted T waves in leads V2-V3, is mainly because of the critical stenosis in the left anterior descending artery (LAD) [15]. Considering TWI's different mechanisms and treatment strategies for these two diseases, it is crucial to differentiate these two entities as soon as possible with minimum facilities such as electrocardiogram.

According to the present study's results, patients with ACS had a more significant TWI sum in the anterior and lateral leads and a larger ratio of the V4/V1 TWI sum. However, they had lower sums in inferior leads and anterior-to-lateral leads TWI ratios. Regarding our results, TWI sum in anterior leads >1.2 mV, in lateral leads >0.9 mV, in anterior-to-inferior leads ratio >12, and in V4/V1 leads ratio >4 are associated with ACS.

Previous studies have suggested that the simultaneous presence of TWI in leads III and V1, described as the “TIIITv1" pattern, suggests acute PE and can differentiate it from ACS [9,11]. Another study reported the association of peak TWI in leads V1-V2 with acute PE [11]. Another study on patients with acute PE and non-ST elevation ACS showed that TWI in leads V1-V3 and inferior leads was predictive for acute PE with high specificity [16]. The specific leads with TWI in patients with ACS and acute PE exhibit differences in their electrophysiological mechanism. TWI in leads III, V1, and V2, commonly observed in acute PE, reflect RV dysfunction. Previous studies revealed that the number of leads presenting TWI is associated with mortality and morbidity in patients with acute PE [17,18]. Another study reported that the sum of TWI amplitudes (≥5 mm) was predictive for acute PE [8]. In this study, we measured the depth of TWI in each lead separately and tried to report the significance of TWI amplitude in each lead as a distinctive factor between acute PE and ACS.

In one study, the total TWI magnitude of the ratio of the anterior-to-inferior leads was more prominent in the ACS group. It also reported the diagnostic value of the V4/V1 ratio in the diagnosis of acute PE with high sensitivity and specificity [12]. These results are consistent with those reported in the present study. We also evaluated whether the depth of TWI (in the anterior/inferior, anterior/lateral, and V4/V1 leads ratios) is associated with the severity of acute PE, and the results showed no association. This finding supports the applicability of the designed ECG-based tool to discriminate between ACS and acute PE since, based on the results, even low-risk acute PE can lead to TWI just as much as the high-risk variant.

In addition to TWI characteristics, we also evaluated the demographic and clinical characteristics of these two groups of patients. The results showed that most patients with ACS had a positive cTnI compared to about one-third of patients with acute PE. Evidence has proven the value of cTnI in determining cardiac injury; however, it cannot define the source or cause of injury and is not exclusive to ACS [19,20]. In patients with acute PE, positive cTnI has been associated with RV dilation, more segmental defects on ventilation-perfusion scintigraphy, and poorer prognosis [21,22]. It is also suggested for risk stratification in these patients [23,24]. The mechanism of cTnI positivity in acute PE is attributed to hypoxemia and hypoperfusion, resulting from the perfusion-ventilation mismatch, low output, and coronary blood flow; a patent foramen oval may lead to paradoxical embolism from systemic veins to the coronary arteries [19]. These results can justify the different rates of cTnI positivity between the patients with ACS and acute PE in our study.

Furthermore, our results showed that most of the patients with acute PE had dyspnea, while chest pain was the dominant symptom in patients with ACS. This result concurs with the current literature [25,26]. It was also obtained from our results that patients with ACS and acute PE were the same age while systolic blood pressure and heart rate were higher among patients with acute PE; however, they were within the normal range and cannot be used to discriminate between the two conditions.

While the gold standard for acute PE diagnosis is CT angiography of pulmonary arteries, these findings imply the clinical value of ECG in differentiation between ACS and acute PE. Previous reports have also referred to the significance of paying greater attention to ECG changes (including ST-T changes, QTc prolongation, arrhythmias, and TWI) for discriminating between MI and PE [27-30]. Regarding our results, the ratio of anterior-to-lateral TWI sum, TWI sum in anterior and lateral leads, and the ratio of anterior-to-inferior in addition to the V4/V1 TWI sum can differentiate ACS from acute PE. Hence, it is suggested that physicians pay more attention to ECG findings in patients suspected of ACS or acute PE.

Limitations

Although this research studied an excellent cohort of patients from three different referral centers, it had some limitations. Since this was a cross-sectional study, we did not follow up with the patients to find the association between the studied indices and the patient's outcomes. The lack of patient follow-up over time and of investigation of their ECG changes, such as reversibility of the TWI, limits the ability to infer causality. All cases were collected from three referral centers in one city. The lack of diversity could affect the generalizability of the findings to other populations or settings.

## Conclusions

In conclusion, acute PE and ACS are two urgent conditions with shared clinical and paraclinical features. Since both may present with sudden-onset chest pain and dyspnea and can elevate cardiac biomarkers, trying to find another diagnostic tool that can facilitate differentiating between these two potentially lethal conditions rapidly seems necessary. Electrocardiogram is a cheap, accessible, and fast diagnostic tool that can be used for the diagnosis. This study found that the TWI sum in anterior leads >1.2 mV, lateral leads >0.9 mV, anterior-to-inferior leads ratio >12, and V4/V1 leads ratio >4 rule out acute PE. Also, the anterior-to-lateral TWI ratio was significantly distinctive among ACS and acute PE patients and can be used as a screening tool. In the emergency setting where CT angiography is unavailable, the anterior-to-lateral TWI ratio can help differentiate between ACS and acute PE. It is recommended that TWI ratios be explored in a larger, more diverse population and examined for their predictive value in different clinical settings.
